# Probiotic and Postbiotic Interactions of *Lactobacillus* Strains with *Candida albicans*: Antifungal Effects Through Microbial Competition

**DOI:** 10.3390/antibiotics15030279

**Published:** 2026-03-10

**Authors:** Andrea Vega-Vásconez, Diana Lucinda Castillo-Patiño, Javier Alberto Garza-Cervantes, Arlette Santacruz, José Rubén Morones-Ramírez

**Affiliations:** 1 Facultad de Ciencias Químicas, Universidad Autónoma de Nuevo León, San Nicolás de los Garza 66455, Mexico; 2 Centro de Investigación en Biotecnología y Nanotecnología, Facultad de Ciencias Químicas, Universidad Autónoma de Nuevo León, Parque de Investigación e Innovación Tecnológica, Apodaca 66628, Mexico; 3 School of Engineering and Science, Tecnologico de Monterrey, Av. Eugenio Garza Sada 2501 Sur, Monterrey 64849, Mexico

**Keywords:** antifungal properties, *Lactobacillus* strains, microbial competition, postbiotic, probiotic

## Abstract

**Background:** *Candida albicans* is the most clinically significant opportunistic fungal pathogen, and the growing resistance to conventional antifungals, particularly azoles and echinocandins, highlights the urgent need for alternative therapeutic strategies. Although lactic acid bacteria (LAB) have shown inhibitory potential against *C. albicans*, the relative contributions of live probiotics, heat-inactivated postbiotics, and cell-free supernatants (CFSs) have rarely been compared in parallel under physiologically relevant conditions against a clinical oral isolate. **Results:** This study systematically evaluated the antifungal activity of *Lactiplantibacillus plantarum* 299V, *Lactobacillus delbrueckii* subsp. *bulgaricus* ATCC 11842, and *Lactobacillus acidophilus* ATCC 4356 using co-culture assays, minimum inhibitory concentration tests, agar well diffusion assays, and optical microscopy. *L. plantarum* achieved the strongest inhibitory effect in co-culture, reducing *C. albicans* viability by 2.39 log_10_ CFU/mL after 24 h, correlating with the greatest acidification of the culture medium. **Methods:** CFS from *L. acidophilus* inhibited fungal growth by 79.01% at native pH, declining to 28.35% upon neutralization to pH 7, confirming that antifungal efficacy is largely pH-dependent and driven by undissociated organic acids. At probiotic concentrations of 1 × 10^9^ CFU/mL, all strains completely suppressed fungal growth. Heat-inactivated postbiotics exhibited up to 95.14% inhibition in MIC assays; however, microscopic analysis revealed coaggregation between postbiotic and fungal cells, which likely interfered with optical density measurements. **Conclusions:** These findings establish that LAB-mediated antifungal activity is multifactorial and assay-dependent, and highlight the importance of distinguishing between probiotic, postbiotic, and CFS effects when developing LAB-based antifungal strategies.

## 1. Introduction

Fungal infections represent a major global health burden, responsible for an estimated 1.7 million deaths each year and disproportionately affecting immunocompromised individuals, including patients with diabetes, cancer, or HIV infection [1]. Among opportunistic fungal pathogens, *Candida albicans* remains the most clinically significant species, responsible for infections that range from superficial mucosal candidiasis to invasive and life-threatening systemic disease [2]. As a polymorphic commensal yeast, *C. albicans* colonizes the oral cavity, gastrointestinal tract, vaginal mucosa, and skin under normal physiological conditions [3]. However, impairment of host immune defenses or disruption of mucosal barriers can promote its transition from commensal organism to invasive pathogen, characterized by filamentation, tissue invasion, and biofilm development [3,4]. The rising prevalence of antifungal resistance, particularly to azoles and echinocandins, together with adverse drug effects and limited therapeutic options, highlights the urgent need for novel and safer antifungal strategies [5].

In this context, LAB, including *Lactiplantibacillus plantarum*, *Lactobacillus acidophilus*, and *Lactobacillus delbrueckii* subsp. *bulgaricus*, have emerged as promising biotherapeutic candidates against *C. albicans* [6,7,8]. These microorganisms are classified as Generally Recognized as Safe and are widely used in fermented foods and probiotic formulations. They naturally colonize gastrointestinal, oral, and urogenital niches [9,10,11]. Beyond their nutritional applications, LAB contribute to mucosal homeostasis through competition with pathogens for nutrients and adhesion sites, reinforcement of epithelial barrier integrity, and modulation of innate and adaptive immune responses [9,11]. Their antifungal activity is mediated by multiple complementary mechanisms, including the production of organic acids, hydrogen peroxide, and bacteriocins that reduce environmental pH and create unfavorable conditions for fungal survival [9]. These metabolites may also interfere with fungal adhesion, morphogenesis, and biofilm formation, thereby enhancing colonization resistance [8,12].

More recently, increasing attention has been directed toward postbiotics, defined as inanimate microorganisms or their bioactive metabolic products, including cell-free supernatants, as alternative therapeutic agents [10]. Postbiotics can exert antimicrobial, immunomodulatory, and anti-inflammatory effects comparable to those of live probiotic cells while reducing potential safety concerns associated with the administration of viable bacteria, particularly in immunocompromised hosts [13]. Despite promising findings, significant knowledge gaps remain regarding the comparative antifungal efficacy of live probiotics, postbiotics, and derived supernatants. Many available studies evaluate single strains, limited experimental endpoints, or reference vaginal isolates of *C. albicans* under simplified laboratory conditions [7,8,14]. Furthermore, strain variability, environmental parameters such as pH and nutrient composition, and time-dependent interaction dynamics complicate mechanistic interpretation.

To address these limitations, the present study systematically investigated the antagonistic interactions of *L. plantarum*, *L. acidophilus*, and *L. delbrueckii* subsp. *bulgaricus* against a clinical oral isolate of *C. albicans*. Using supplemented MRS broth as a competitive model system, inhibition dynamics were characterized through coculture assays, quantitative viable cell enumeration, pH measurement, and microscopic analysis. The antifungal potential of native and pH-neutralized cell-free supernatants, as well as heat-inactivated postbiotic preparations, was further evaluated using agar diffusion and minimum inhibitory concentration assays. By systematically comparing probiotics, postbiotics, and CFS under physiologically relevant conditions, this study provides a mechanistic framework for understanding strain-specific LAB–*Candida* interactions and offers a comparative foundation for the rational design of probiotic and postbiotic-based antifungal interventions.

## 2. Results and Discussion

### 2.1. Co-Culture

**Figure 1:**
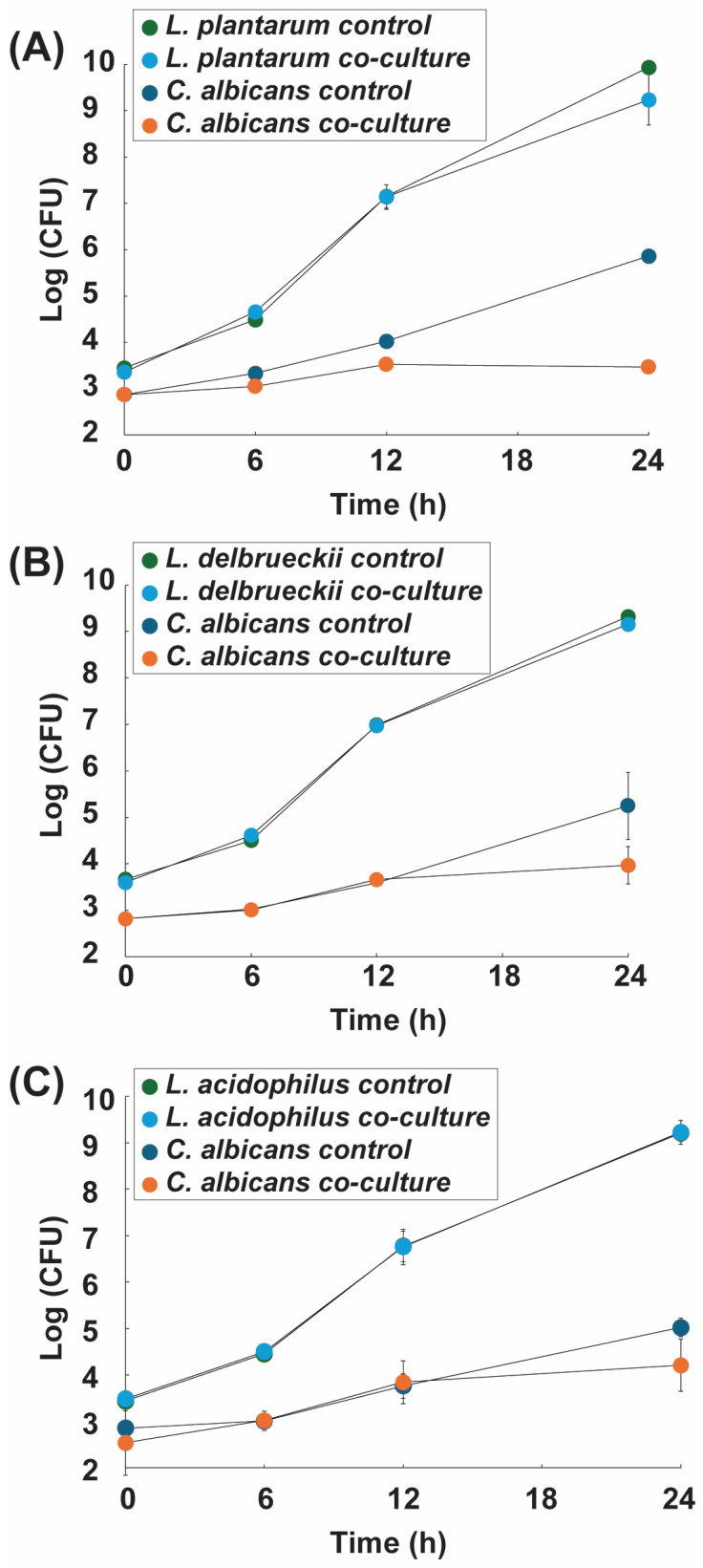
Growth curves of lactic acid bacteria (LAB) and *Candida albicans* in monoculture and co-culture. (**A**) *Lactiplantibacillus plantarum*, (**B**) *Lactobacillus delbrueckii* subsp. *bulgaricus*, and (**C**) *Lactobacillus acidophilus* were grown individually (control) or in co-culture with *C. albicans*. Cell densities are expressed as log_10_ CFU/mL over 24 h. LAB maintained robust growth in co-culture, while *C. albicans* exhibited reduced viability, with the strongest inhibition observed in the presence of *L. plantarum*. Error bars represent the standard deviation of triplicate experiments.

The inhibitory activity of LAB strains against *Candida albicans* was evaluated in co-culture over 24 h (Figure 1A–C). Growth curves revealed that *C. albicans* growth was markedly reduced in the presence of LAB, with inhibition becoming evident after 12 h. By 24 h, *Lactiplantibacillus plantarum* achieved the strongest effect, reducing *C. albicans* viability by 2.39 log_10_ CFU/mL, corresponding to approximately 99% decrease in fungal viability, followed by *Lactobacillus delbrueckii* (1.28 log_10_ CFU/mL) and *Lactobacillus acidophilus* (0.81 log_10_ CFU/mL). The reductions observed for *L. delbrueckii* and *L. acidophilus* fall within the 1–2 log_10_ range considered biologically relevant probiotic- Candida interaction models [15]. In contrast, all LAB strains maintained robust growth in co-culture, with final cell densities comparable to their monoculture controls [16,17]. The reductions in *C. albicans* viability observed in co-culture were statistically significant compared with monoculture controls (one-way ANOVA followed by Tukey’s post hoc test, *p* < 0.05).

This inhibitory effect coincided with a progressive decline in culture pH, which reached 3.94 for *L. plantarum*, 4.27 for *L. delbrueckii*, and 4.31 for *L. acidophilus* after 24 h (Figure S1A–C). Acidification likely enhanced antifungal activity by facilitating the diffusion of undissociated lactic acid across the *C. albicans* cell membrane, leading to intracellular acidification, disruption of homeostasis, and increased sensitivity to oxidative stress [10,18]. Under acidic conditions, *C. albicans* activates *RIM101*-dependent transcriptional responses and stress regulators such as *HOG1*, which are required for adaptation to low-pH environments [19,20]. Sustained acid stress increases the energetic demand needed to maintain intracellular pH homeostasis, thereby limiting fungal growth [21]. This growth limitation is consistent with activation of pH-stress regulatory pathways and the increased metabolic burden required to preserve intracellular pH balance under weak-acid conditions, collectively constraining fungal proliferation [19,20,21].

Although our study did not directly assess gene expression, previous work has demonstrated that *L. plantarum* in co-culture with *C. albicans* upregulates genes linked to environmental sensing and adhesion, while *C. albicans* downregulates virulence-associated genes, including those linked to hyphal formation and adhesion, such as ALS3, HWP1, CPH1, and BCR1. These findings suggest that beyond acidification, competitive interactions may involve transcriptional reprogramming in both organisms, consistent with the stronger inhibitory effect observed for *L. plantarum* [8]. These findings highlight that antifungal inhibition in co-culture likely involves direct competition and localized metabolic effects, which may not be fully captured by diffusion-based solid assays.

### 2.2. Agar Well Diffusion

#### 2.2.1. Inhibition from Postbiotic

**Figure 2:**
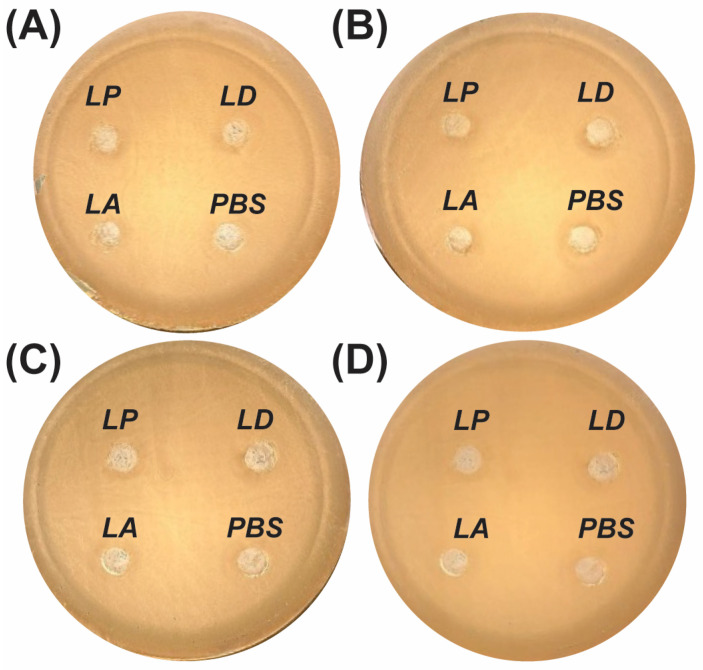
Agar well diffusion assay evaluating the antifungal activity of lactic acid bacteria (LAB) and their derivatives against *Candida albicans*. (**A**) Postbiotics, (**B**) live LAB, (**C**) cell-free supernatants (CFSs), and (**D**) neutralized CFS (pH 7) were tested. Wells contained *Lactiplantibacillus plantarum* (LP), *Lactobacillus delbrueckii* (LD), *Lactobacillus acidophilus* (LA), or phosphate-buffered saline (PBS, negative control). No clear inhibition zones were observed under the tested conditions.

No inhibition zones were detected when postbiotics derived from *L. plantarum*, *L. delbrueckii*, or *L. acidophilus* were tested against *C. albicans* (Figure 2A). Previous studies have reported inhibition zones of 11.5–16.5 mm against several bacterial strains using heat-inactivated *L. acidophilus* IFFI 6005 [22]. Similarly, heat-inactivated *L. delbrueckii* subsp. *bulgaricus* NCDO 2394 produced inhibition zones against *E. coli* and *K. pneumoniae* [23]. These discrepancies likely reflect key methodological differences between studies, including the target microorganism (bacterial strains vs. *C. albicans*), fermentation scale and postbiotic concentration steps, heat-inactivation protocols, agar composition and well diameter, inoculum density, and incubation conditions. Such variables can substantially influence diffusion-based inhibition outcomes.

#### 2.2.2. Inhibition from Live LAB

Consistent with the postbiotic results, no inhibition of *C. albicans* by live LAB strains was observed in our agar well diffusion assays (Figure 2B). This finding differs from the literature, where inhibition halos of 6–10 mm were observed for *L. plantarum* using the overlay method [8]. Likewise, other research groups have observed a 19 mm inhibition zone with *L. paracasei* 28.4 [17]. However, Yocheva et al. reported that even different strains within the same species may not exhibit inhibitory activity against the same *C. albicans* strain [7]. Since comparable overlay methodologies were employed across studies, strain-dependent variability in antimicrobial metabolite production most likely accounts for the absence of inhibition observed in the present study.

#### 2.2.3. Inhibition from CFS and Neutralized CFS (pH 7)

Neither the native nor the pH-neutralized cell-free supernatants (CFSs) from *L. plantarum*, *L. delbrueckii*, or *L. acidophilus* exhibited inhibitory activity against *C. albicans* in the agar diffusion assay (Figure 2C,D). Previous studies have similarly reported limited or absent antifungal activity of LAB-derived CFS when assessed using diffusion-based methods [24]. LAB-derived CFS are known to contain diverse bioactive metabolites—including organic acids, hydrogen peroxide, phenyllactic acid, and, in some strains, bacteriocin-like compounds—that may contribute to antifungal effects under appropriate conditions [25,26]. However, the agar well diffusion method presents inherent limitations, particularly for slowly diffusing or high–molecular-weight molecules, which may fail to reach inhibitory concentrations at the fungal growth front [17,27,28]. Because the CFS was not chemically fractionated or metabolically characterized in the present study, the discussion of specific metabolites remains inferential and literature-based. The inhibitory activity observed in liquid-phase MIC assays, therefore, supports the interpretation that the absence of inhibition zones in agar diffusion reflects methodological constraints rather than the absence of antifungal potential.

### 2.3. Minimum Inhibitory Concentration

#### 2.3.1. Anti-Candida Activity of CFS

**Figure 3:**
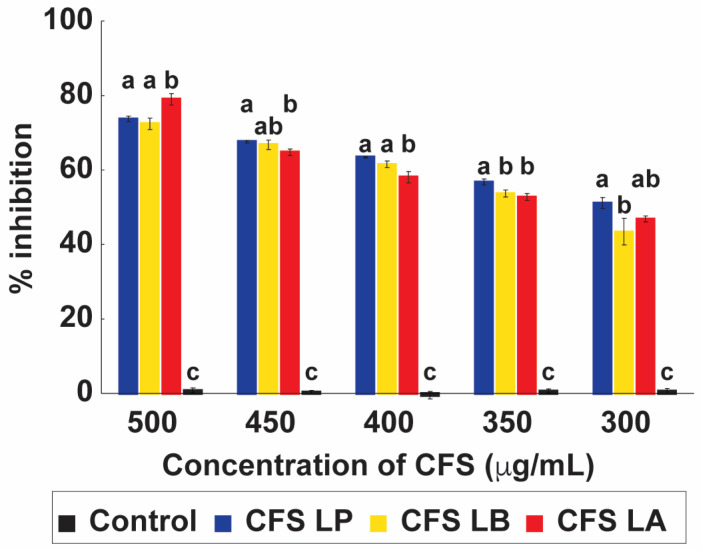
Dose-dependent antifungal activity of cell-free supernatants (CFSs) from lactic acid bacteria (LAB) against *Candida albicans*. CFSs from *Lactiplantibacillus plantarum* (LP), *Lactobacillus delbrueckii* subsp. *bulgaricus* (LB), and *Lactobacillus acidophilus* (LA) were tested at concentrations ranging from 300 to 500 µg/mL. Growth inhibition of *C. albicans* is expressed as a percentage relative to the untreated control. Bars represent mean ± SD (*n* = 3). Different letters above the bars denote statistically significant differences between treatments (*p* < 0.05), while identical letters indicate no significant difference.

The cell-free supernatants (CFSs) from *L. plantarum*, *L. delbrueckii*, and *L. acidophilus* all inhibited *C. albicans* growth in a dose-dependent manner across the concentration range of 500–300 µg mL^−1^ (Figure 3). At the highest concentration (500 µg mL^−1^), CFS from *L. acidophilus* exhibited the strongest inhibition (79.01%), followed by *L. plantarum* (73.72%) and *L. delbrueckii* (72.41%). Although inhibitory activity declined with decreasing concentrations, significant inhibition was still observed at 300 µg mL^−1^ for all three strains (*p* < 0.05). Statistical analysis confirmed that differences between treatments were strain- and concentration-dependent.

These results are in line with earlier reports, such as the 74.92% inhibition documented for *L. plantarum* CFS against *C. albicans*, though strain-specific variability in antifungal activity has been widely noted [8]. The observed inhibition is likely attributable to organic acids, particularly lactic acid, which are abundant metabolites in LAB supernatants. Supporting this interpretation, prior studies demonstrated that lactic acid at 200 mM reduces both hyphal formation and viability in *C. albicans*, underscoring its dual role in limiting fungal morphology and survival [29]. Importantly, these effects depend on an acidic environment, as undissociated lactic acid can diffuse across the fungal membrane more effectively under low pH, leading to intracellular acidification and loss of homeostasis [30]. Collectively, these findings reinforce the role of LAB-derived organic acids as central mediators of antifungal activity in CFS preparations.

#### 2.3.2. Anti-Candida Activity of CFS pH 7

**Figure 4:**
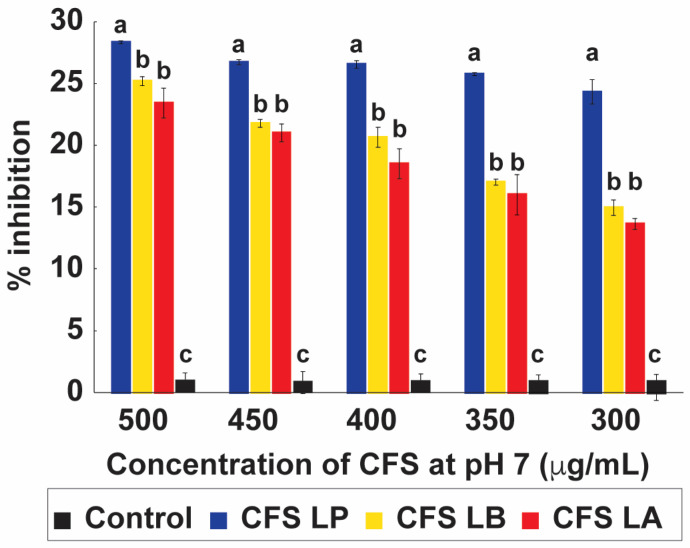
Antifungal activity of pH-neutralized cell-free supernatants (CFSs) from lactic acid bacteria (LAB) against *Candida albicans*. CFS from *Lactiplantibacillus plantarum* (LP), *Lactobacillus delbrueckii* subsp. *bulgaricus* (LB), and *Lactobacillus acidophilus* (LA) were adjusted to pH 7 and tested at concentrations ranging from 300 to 500 µg/mL. Growth inhibition of *C. albicans* is expressed as a percentage relative to the untreated control. Bars represent mean ± SD (*n* = 3). Different letters above the bars denote statistically significant differences between treatments (*p* < 0.05), while identical letters indicate no significant difference.

The pH-neutralized CFS from *L. plantarum*, *L. delbrueckii*, and *L. acidophilus* retained significant inhibitory activity against *C. albicans* across all concentrations tested (500–300 µg mL^−1^), with maximum inhibition values of 28.35%, 25.21%, and 23.43%, respectively (Figure 4). Although activity was lower than that observed under acidic conditions (Figure 3), inhibition remained consistent and strain-dependent. These results are comparable to the literature, where inhibition of approximately 33% for *L. fermentum* 18 A-TV when its CFS was adjusted to pH 7.5 has been observed [18].

The reduced antifungal effect at neutral pH likely reflects the dissociation state of lactic acid, one of the principal metabolites produced by LAB. Under acidic conditions, lactic acid remains largely undissociated, enabling efficient diffusion across the fungal cell membrane [11,31,32]. Once inside the cell, it dissociates, acidifying the cytoplasm and disrupting homeostasis. At neutral pH, however, lactic acid exists predominantly in its dissociated form, which is less membrane-permeable, thereby diminishing its antifungal efficacy [10,18].

#### 2.3.3. Anti-Candida Activity of Probiotics

**Figure 5:**
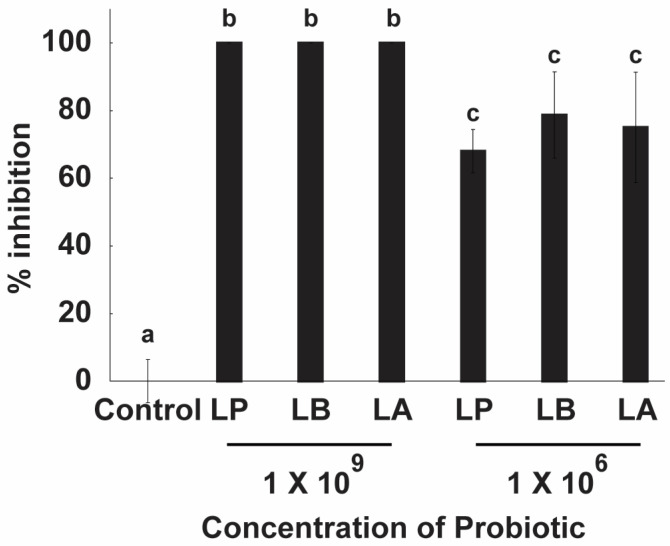
Antifungal activity of probiotic lactic acid bacteria (LAB) against *Candida albicans* in co-culture. *Lactiplantibacillus plantarum* (LP), *Lactobacillus delbrueckii* (LB), and *Lactobacillus acidophilus* (LA) were tested at two concentrations (1 × 10^9^ and 1 × 10^6^ CFU/mL) after 24 h of co-culture with *C. albicans*. Growth inhibition is expressed as a percentage relative to the untreated control. Complete inhibition was observed at the higher concentration, while partial inhibition was maintained at the lower concentration in a strain-dependent manner. Bars represent mean ± SD (*n* = 3). Different letters above the bars denote statistically significant differences between treatments (*p* < 0.05), while identical letters indicate no significant difference.

As shown in Figure 5, all probiotic strains—*L. plantarum*, *L. delbrueckii*, and *L. acidophilus*—completely inhibited *C. albicans* growth at a concentration of 1 × 10^9^ CFU/mL. At a lower concentration of 1 × 10^6^ CFU/mL, inhibition was reduced but remained significant, with *L. plantarum* achieving 68.09%, *L. delbrueckii* 78.72%, and *L. acidophilus* 75.04%. These findings indicate that higher probiotic densities are required for full suppression of fungal growth, while lower densities result in moderate and strain-dependent inhibition, with *L. delbrueckii* showing the greatest efficacy under reduced concentrations.

Our results differ from the literature, where a 1.5 log-cycle inhibition has been observed using *L. plantarum* at 1 × 10^8^ CFU/mL in mixed culture with *C. albicans* and *S. mutans* at 1 × 10^3^ CFU/mL. The lower inhibition observed in their study may be related to the growth medium (TSBYE) and the additional presence of *S. mutans* [29]. Conversely, our findings are consistent with research groups that have observed 82.34% inhibition after 24 h of co-culturing *L. plantarum* with *C. albicans* in MRS medium supplemented with yeast extract, peptone, and dextrose (YDP) [8].

The inhibitory effect of probiotics appears closely linked to the ratio of LAB to *C. albicans* cells. Yeast cells (≈6 µm) are considerably larger than bacterial cells (≈1.5 µm), and a higher bacterial density increases the likelihood of coaggregation, enabling LAB to physically cover and interact with fungal cells. This direct interaction enhances both nutrient competition and the local delivery of antifungal metabolites, including organic acids, bacteriocins, biosurfactants, and hydrogen peroxide [33].

Nutrient competition also plays a critical role. LAB have shorter generation times and rapidly deplete essential nutrients such as glucose, thereby restricting availability for *C. albicans*. This depletion not only limits fungal growth but also alters the culture environment by lowering pH, generating conditions that are less favorable for yeast development and virulence [10,34]. Collectively, these results highlight that both cell density and metabolic competition are central to LAB-mediated inhibition of *C. albicans*.

#### 2.3.4. Anti-Candida Activity of Postbiotics

**Figure 6:**
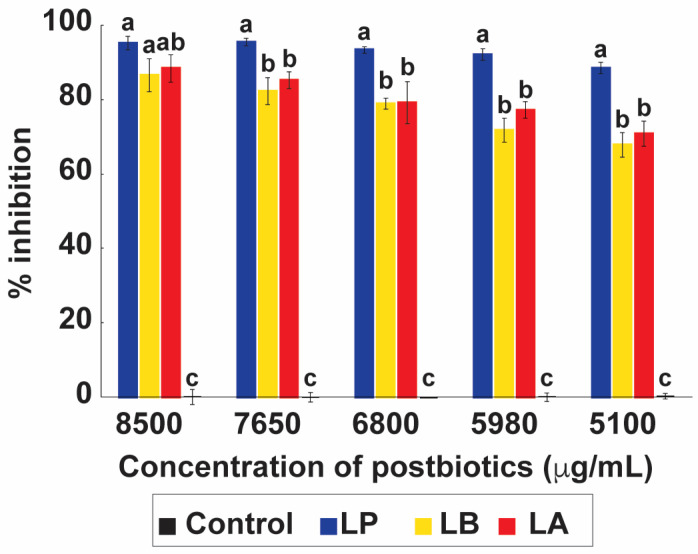
Antifungal activity of postbiotics derived from lactic acid bacteria (LAB) against *Candida albicans*. Postbiotics prepared from *Lactiplantibacillus plantarum* (LP), *Lactobacillus delbrueckii* (LB), and *Lactobacillus acidophilus* (LA) were tested at concentrations ranging from 5100 to 8500 µg/mL. Growth inhibition of *C. albicans* is expressed as a percentage relative to the untreated control. Bars represent mean ± SD (*n* = 3). Different letters above the bars denote statistically significant differences between treatments (*p* < 0.05), while identical letters indicate no significant difference.

The postbiotics derived from *L. plantarum* (LP), *L. delbrueckii* (LB), and *L. acidophilus* (LA) all exhibited a clear concentration-dependent inhibitory effect against *C. albicans* (Figure 6). At the highest concentration tested (8500 µg mL^−1^), inhibition reached 95.14% for LP, 88.35% for LA, and 86.50% for LB. Although inhibition declined with decreasing concentrations, substantial antifungal activity was still observed at 5100 µgmL^−1^, with LP maintaining 88.43% inhibition. These findings suggest concentration-dependent inhibitory activity in OD-based MIC assays; however, this effect should be interpreted with caution, given the potential interference of coaggregation with optical density measurements. Comparable results have been reported in previous studies. For example, Miao et al. showed that heat-inactivated *L. acidophilus* IFFI 6005 displayed measurable antimicrobial effects against bacterial pathogens, while Van et al. demonstrated that postbiotics from *L. delbrueckii* inhibited *E. coli* and *K. pneumoniae* [22,23]. Although most of these reports focus on antibacterial activity, our results extend these observations to antifungal activity against *C. albicans*, highlighting the broader therapeutic potential of postbiotic preparations.

The high inhibition observed with postbiotics in our study may be attributed to multiple factors, including the persistence of bioactive cell wall components (such as S-layer proteins, peptidoglycan, or lipoteichoic acids) and the presence of heat-stable metabolites. Prior studies have shown that S-layer proteins from LAB mediate microbial adhesion and coaggregation [35], processes that could facilitate close interactions with fungal cells even after bacterial inactivation. Furthermore, organic acids and other metabolites may remain active after heat treatment, providing continued antifungal effects [36].

Together, these findings suggest that postbiotic antifungal activity may be assay-dependent and partly indirect, as high inhibition values observed in OD-based MIC assays were not reproduced in co-culture or agar diffusion assays, likely due to coaggregation-mediated interference with spectrophotometric readings. Therefore, the postbiotic data support inhibitory potential under the tested conditions, but do not by themselves establish strong direct antifungal activity. Given their stability and safety advantages over live probiotics, LAB-derived postbiotics represent a promising strategy for antifungal interventions, especially in immunocompromised hosts where live probiotic use may be contraindicated.

#### 2.3.5. Postbiotics in Coculture Assays

**Figure 7:**
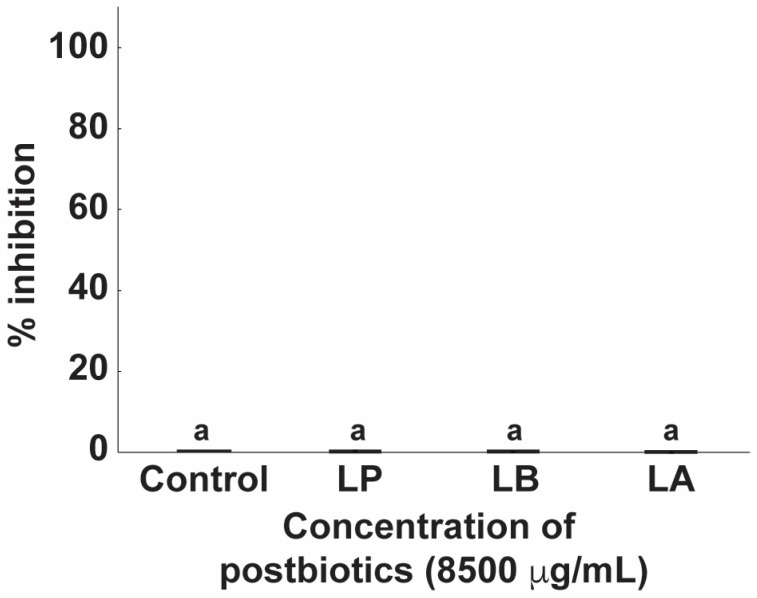
Antifungal activity of postbiotics in co-culture assays with *Candida albicans*. Postbiotics from *Lactiplantibacillus plantarum* (LP), *Lactobacillus delbrueckii* (LB), and *Lactobacillus acidophilus* (LA) were tested at 8500 µg/mL during 24 h co-culture in fortified MRS medium. No significant inhibition of *C. albicans* growth was observed compared with the control. Bars represent mean ± SD (*n* = 3). Identical letters above the bars indicate no statistically significant differences between treatments (*p* ≥ 0.05).

**Figure 8:**
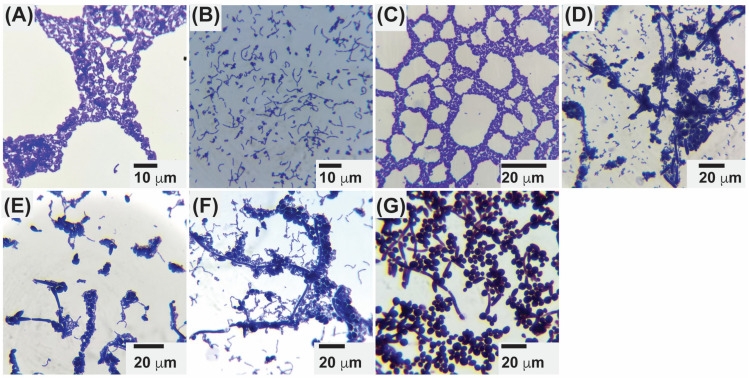
Microscopic evaluation of postbiotics and their interaction with *Candida albicans*. Representative crystal violet–stained images (100× magnification) show postbiotics from (**A**) *Lactiplantibacillus plantarum*, (**B**) *Lactobacillus delbrueckii*, and (**C**) *Lactobacillus acidophilus* alone, as well as their co-cultures with *C. albicans* (**D**–**F**). Panel (**G**) shows *C. albicans* alone as a control. Coaggregation between postbiotics and fungal cells is evident in mixed samples (**D**–**F**), where clusters of LAB postbiotic material associate closely with *C. albicans* hyphae and yeast cells.

In contrast to the strong antifungal activity observed in the MIC assay (Figure 6), no significant inhibition of *C. albicans* was detected in coculture at the same highest postbiotic concentration (8500 µg/mL; *p* > 0.05) (Figure 7). This discrepancy may be explained by coaggregation between postbiotics and *C. albicans*, as observed microscopically (Figure 8). Coaggregation can alter optical density (OD) readings by reducing light scattering relative to individual cells, potentially masking true inhibition when using spectrophotometric methods. Although OD values are commonly used to estimate CFU/mL in microplate assays, the correlation can be influenced by factors such as cell density, size, and light path length [37]. Thus, our results suggest that the lack of measurable inhibition in coculture may reflect methodological limitations rather than the absence of biological activity.

The discrepancy between the MIC and agar well diffusion results can be attributed to fundamental differences between liquid and solid culture systems. In MIC assays, antifungal metabolites are uniformly distributed and maintain continuous contact with *C. albicans*, allowing consistent exposure. In contrast, in solid agar, compound distribution is spatially restricted, generating concentration gradients that may limit the effective dose reaching fungal cells [38]. Furthermore, pH changes in solid media tend to remain localized around the colony rather than being evenly distributed as in liquid cultures [39,40]. Additionally, lactobacilli can co-aggregate with pathogenic microorganisms, creating localized microenvironments enriched in inhibitory substances [7]. Together, these physicochemical and biological factors may explain why inhibition was observed in MIC assays but not in agar well diffusion tests.

### 2.4. Microscopic Evaluation of Postbiotic–C. albicans Interactions

Microscopic analysis confirmed clear coaggregation between postbiotics from *L. plantarum*, *L. delbrueckii*, *L. acidophilus*, and *C. albicans* after 24 h of incubation (Figure 8). These findings are consistent with the literature, where coaggregation of *L. plantarum* and *L. acidophilus* has been observed with *C. albicans* [41], and with other studies reporting *L. delbrueckii* interactions with *Saccharomyces cerevisiae* [7].

Coaggregation in LAB is thought to be mediated primarily by S-layer proteins, which interact with microbial cell wall components such as lipids and teichoic acids [35,42]. Removal of these proteins significantly reduces adhesion and coaggregation [43], while S-layer–associated proteins (SLAPs) further contribute to these interactions [44]. Notably, these proteins are highly stable and capable of self-assembly even after heat stress. In our study, probiotics were exposed to 121 °C at 15 psi for 15 min, yet retained coaggregation capacity, suggesting that S-layer proteins remained functional [45].

On the fungal side, *C. albicans* is also capable of coaggregation through adhesins such as *ALS3* and *HWP1*, which mediate interactions with oral bacteria, including *Porphyromonas gingivalis* and *Streptococcus mutans* [46]. Thus, the interactions observed here likely result from the combined contribution of both LAB and *C. albicans*.

While these physical interactions did not translate into measurable inhibition in coculture assays, they may nonetheless play a role in shaping host–microbe or microbe–microbe dynamics in vivo. Previous work has shown that postbiotics can activate immune receptors and modulate cytokine responses [33]. Although not addressed in the present study, such effects highlight the potential broader benefits of LAB-derived postbiotics beyond direct antifungal activity.

### 2.5. Strengths and Limitations

Although working with a single isolate naturally limits the generalizability of the findings, the use of a clinical oral isolate of *C. albicans* represents a notable strength of this study. Unlike standardized reference strains, clinical isolates better reflect the virulence factors, resistance profiles, and adaptive capacity of strains encountered in real patient settings [47,48]. Furthermore, the oral cavity represents an underexplored niche in the context of LAB–*C. albicans* interactions, as most published work has focused on vaginal or gastrointestinal isolates, making the present study one of the first to evaluate these interactions using an oral clinical strain [49,50,51]. This distinction is relevant given that oral candidiasis presents unique ecological and immunological conditions that differ substantially from other mucosal sites. That said, direct comparison with the existing literature was not always straightforward, and this challenge was further compounded by the fact that, at the time this study was initiated, data on co-culture interactions between *C. albicans* and LAB were still scarce. While the field has since advanced, certain mechanistic questions remain open [7,8,52]. Although this study offers a comprehensive comparative evaluation through co-culture assays, agar well diffusion, MIC tests, and coaggregation assessment, the specific metabolites responsible for the observed inhibition and the genes involved in LAB–*C. albicans* interactions were not directly characterized [53,54]. Addressing these molecular aspects through metabolomic and transcriptomic approaches, combined with validation across a broader panel of clinical isolates, represents an important direction for future research.

## 3. Experimental Section

### 3.1. Microorganisms and Coculture Conditions

**Figure 9:**
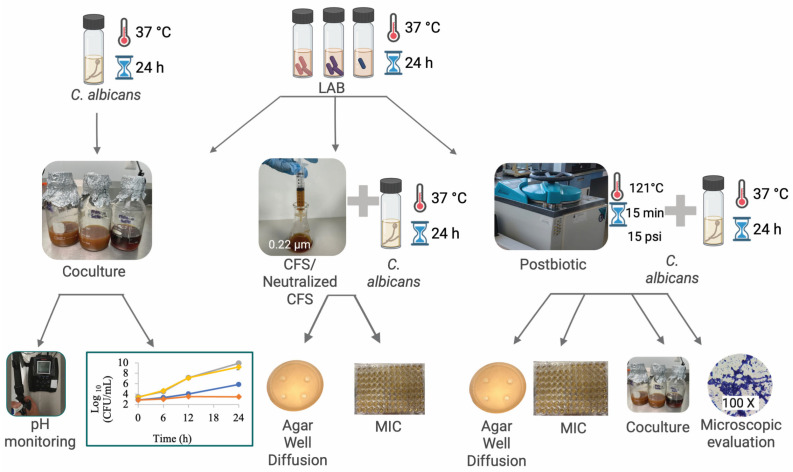
Schematic representation of the experimental design. Candida albicans and three lactic acid bacteria (LAB) strains (*Lactiplantibacillus plantarum*, *Lactobacillus delbrueckii* subsp. *bulgaricus*, and *Lactobacillus acidophilus*) were cultured at 37 °C for 24 h. Three experimental strategies were evaluated: (1) co-culture assays, in which LAB and *C. albicans* were grown together with monitoring of pH and microbial growth dynamics; (2) cell-free supernatants (CFSs) and neutralized CFS, obtained by filtration (0.22 µm) and tested against *C. albicans* using agar well diffusion and minimum inhibitory concentration (MIC) assays; and (3) postbiotic preparations, generated by heat inactivation (121 °C, 15 psi, 15 min) and evaluated by agar diffusion, MIC, co-culture assays, and microscopic analysis of coaggregation.

An overview of the experimental workflow, including the integration of co-culture, CFS/neutralized CFS, and postbiotic assays, is presented in Figure 9. Three probiotic strains *L. plantarum* 299v (DSM 9843, Deutsche Sammlung von Mikroorganismen und Zellkulturen, Braunschweig, Germany), *Lactobacillus delbrueckii* subsp. *bulgaricus* (ATCC 11842, American Type Culture Collection, Manassas, VA, USA), and *Lactobacillus acidophilus* (ATCC 4356, American Type Culture Collection, Manassas, VA, USA)—were obtained from the Nutrionomics Research Group at Tecnológico de Monterrey. We selected the species *Lactiplantibacillus plantarum*, *Lactobacillus acidophilus*, and *Lactobacillus delbrueckii* subsp. *bulgaricus* as representative, widely used probiotic LAB with documented anti-*Candida* potential and relevance to oral/gastrointestinal niches, offering complementary functional traits (e.g., organic-acid production, bacteriocin-like activity, and cell-surface adhesion/coaggregation). *L. plantarum* 299v was selected due to its demonstrated capacity to reduce pathogenic bacteria in the oropharynx and its high production of lactic acid [55,56]. *L. acidophilus* ATCC 4356 was included based on its inhibitory activity against *C. albicans* biofilm formation and filamentation, as well as its production of acidocin 4356, a multifunctional bacteriocin with anti-infective capacity through inhibition of virulence factors and biofilm degradation [55,57]. *L. delbrueckii* was chosen given evidence of its inhibitory activity against *Candida* spp. through the production of bacteriocins and cell-free supernatants with demonstrated antifungal effects [58]. A clinical oral isolate of *C. albicans* was provided by Hospital San Vicente (Monterrey, Mexico) and was identified through CHROMagar™ Candida and VITEK^®^ 2 (BioMérieux, Marcy-l’Étoile, France) systems. The isolate was recovered from an oral clinical sample and stored at −70 °C in glycerol stocks; all experiments were performed using freshly revived cultures within five subcultures from the original stock to minimize phenotypic drift and adaptive variation.

Cocultures were established as described in Table S1. Each strain was activated in its respective growth medium (MRS for LAB species and YM for *C. albicans*) and incubated at 37 °C for 24 h. The resulting cell pellets were harvested by centrifugation (10,000× *g* for 10 min), washed three times with phosphate-buffered saline (PBS, pH 7.2), and resuspended in a PBS-glycerol solution (18 mL PBS and 2 mL glycerol). The cell suspensions were stored at −70 °C for subsequent use.

For coculture preparation, the activated cultures were added to 250 mL of supplemented MRS broth (Table S2), where each LAB strain was inoculated at 1 × 10^3^ CFU mL^−1^, along with *C. albicans* at 1 × 10^2^ CFU mL^−1^. These inoculum levels were selected based on previously published LAB–*C. albicans* interaction models [59]. Higher starting densities (e.g., ≥10^7^ CFU mL^−1^ were avoided because they compress logarithmic growth and reduce the sensitivity of kinetic comparisons [8]. Monocultures of LAB and *C. albicans* were used as controls, each inoculated at the same concentrations. The cultures were incubated at 37 °C for 24 h, and 30 mL aliquots were collected every 6 h to measure pH, titratable acidity, and microbial growth.

Microbial growth was assessed by plate counting. Serial dilutions of the cultures or cocultures were prepared in buffered peptone water (0.1% bacteriological peptone), and 100 µL aliquots were plated on the appropriate agar. LAB growth in coculture was evaluated on MRS agar with fluconazole (Best Laboratories, 2 mg mL^−1^) to inhibit *C. albicans*, while *C. albicans* growth was assessed on YM agar with erythromycin (Alpharma Laboratories, 1 mg mL^−1^) to inhibit LAB. Control cultures were plated on MRS agar for LAB and YM agar for *C. albicans*, cultured without antibiotics. Prior to coculture experiments, we verified that fluconazole (2 mg mL^−1^) did not affect LAB recovery on MRS agar and that erythromycin (1 mg mL^−1^) did not affect *C. albicans* recovery on YM agar, ensuring selective enumeration.

The titration of acidity was performed by transferring 10 mL of the culture or coculture to a 250 mL Erlenmeyer flask, followed by the addition of 50 mL of distilled water to ensure proper dilution. Two to three drops of phenolphthalein were added as an indicator, and the solution was titrated with a 1 N NaOH standard solution, added dropwise while stirring continuously. The endpoint was reached when the solution turned a persistent pink color for 30 s, indicating neutralization of the acids in the sample. Lactic acid percentage was calculated using the formula:

$$\%\;lactid\;acid=\left(\frac{V_{2}\times N\times mEq}{V_{1}}\right)\times 100$$

V1 = Sample volume;

V2 = NaOH volume used;

N = NaOH normality;

MEq = 0.090 g of lactic acid.

### 3.2. Preparation of Cell-Free Supernatants (CFSs)

Each LAB strain was cultured in MRS, then centrifuged (10,000× *g*, 10 min), and the supernatant was filtered (0.22 µm). For neutralized CFS (pH 7), pH was adjusted with NaOH (8 M) before filtration. CFS samples were stored at 5 °C.

### 3.3. Postbiotic Production

The LAB was prepared as above, autoclaved at 121 °C and 15 psi for 15 min, and resuspended in YM medium at 1.7% (*w v*^−1^). Viability was confirmed by plating.

### 3.4. Antifungal Activity Assays

#### 3.4.1. Agar Well Diffusion

Following Yocheva et al. (2024) [7] with modifications, double-layer YM agar was inoculated with *C. albicans* (1 × 10^7^ CFU mL^−1^), and 6 mm wells were filled with 100 µL of CFS (at native pH or adjusted to pH 7), postbiotics, or LAB suspensions. Plates were incubated at 37 °C for 24 h.

#### 3.4.2. Minimum Inhibitory Concentration

Serial dilutions of antifungal agents (CFS, CFS pH 7, postbiotics) were tested in 96-well microplates at concentrations ranging from 500 to 3000 µg mL^−1^ for CFS and CFS pH 7, and from 8500 to 5100 µg mL^−1^ for postbiotics, in combination with *C. albicans* (1 × 10^6^ CFU mL^−1^). After 24 h of incubation at 37 °C, OD was measured. For postbiotic evaluation, viable counts were performed at 0 and 24 h using serial dilutions and YM agar plating [46]. For probiotic evaluation, LAB strains were mixed at concentrations of 1 × 10^6^ CFU mL^−1^ or 1 × 10^9^ CFU mL^−1^ with *C. albicans* (1 × 10^6^ CFU mL^−1^), followed by 24 h of incubation at 37 °C. Growth was then evaluated for both the control and the co-culture with LAB.

#### 3.4.3. Microscopic Evaluation

To evaluate the potential coaggregation between postbiotics derived from postbiotics of LAB and *C. albicans*, staining was performed using mixed cultures of both organisms. Initially, the samples were heat-fixed onto glass slides, followed by the application of crystal violet for 1 min. The slides were then washed with distilled water and refixed by heat. Finally, the samples were examined under an optical microscope at 100× magnification using mineral oil [7].

### 3.5. Statistical Analysis

In this study, all treatments and controls were performed in triplicate, with each treatment spaced over time to allow for proper assessment. Data distribution was assessed for normality using the Shapiro–Wilk test prior to statistical analysis. Because the primary objective was to compare treatments within each fixed experimental setting (e.g., among strains at a given concentration/dose within a given assay), groups were defined per assay as the set of strain–concentration conditions compared in that experiment. Accordingly, statistical significance was evaluated using one-way analysis of variance (ANOVA) followed by Tukey’s HSD post hoc test (95% confidence interval) [7]. A *p*-value < 0.05 was considered statistically significant. While a two-way ANOVA can be used to test main effects and interaction terms (strain × concentration), formal interaction testing was not the primary objective of this work.

## 4. Conclusions

This study demonstrates that LAB exert antifungal activity against *C. albicans* through complementary mechanisms, including direct competitive inhibition in co-culture, pH-dependent CFS activity driven by undissociated organic acids, and coaggregation by heat-inactivated postbiotics. Notably, postbiotic inhibition observed in OD-based MIC assays was not confirmed in co-culture or agar diffusion assays, likely reflecting coaggregation-mediated interference rather than direct antifungal activity. Future research should elucidate the molecular mechanisms underlying LAB–*Candida* interactions and explore the immunomodulatory potential of postbiotics, supporting the development of LAB-based strategies to combat *Candida* infections and antifungal resistance.

## Data Availability

The original contributions presented in this study are included in the article. Further inquiries can be directed to the corresponding author.

## Supplementary Materials

The following supporting information can be downloaded at https://www.mdpi.com/article/10.3390/antibiotics15030279/s1. Figure S1. Acidity (% lactic acid) and pH of *C. albicans* vs. BAL. (A) *L. plantarum* vs. *C. albicans* (B) *L. delbrueckii* vs. *C. albicans* (C) *L. acidophilus* vs. *C. albicans.* Table S1. Coculture of *C. albicans* and *L. plantarum*, *L. delbrueckii*, *L. acidophilus*. Table S2: Components of the Supplemented MRS.

## References

[1] Kainz K., Bauer M.A., Madeo F., Carmona-Gutierrez D. (2020). Fungal Infections in Humans: The Silent Crisis. MIC.

[2] Nikou S.-A., Kichik N., Brown R., Ponde N., Ho J., Naglik J., Richardson J. (2019). *Candida albicans* Interactions with Mucosal Surfaces during Health and Disease. Pathogens.

[3] Talapko J., Juzbašić M., Matijević T., Pustijanac E., Bekić S., Kotris I., Škrlec I. (2021). *Candida albicans*—The Virulence Factors and Clinical Manifestations of Infection. JoF.

[4] Pappas P.G., Lionakis M.S., Arendrup M.C., Ostrosky-Zeichner L., Kullberg B.J. (2018). Invasive Candidiasis. Nat. Rev. Dis. Primers.

[5] Soriano A., Honore P.M., Puerta-Alcalde P., Garcia-Vidal C., Pagotto A., Gonçalves-Bradley D.C., Verweij P.E. (2023). Invasive Candidiasis: Current Clinical Challenges and Unmet Needs in Adult Populations. J. Antimicrob. Chemother..

[6] Huang Z., Zhu J., Bu X., Lu S., Luo Y., Liu T., Duan N., Wang W., Wang Y., Wang X. (2025). Probiotics and Prebiotics: New Treatment Strategies for Oral Potentially Malignant Disorders and Gastrointestinal Precancerous Lesions. npj Biofilms Microbiomes.

[7] Yocheva L., Tserovska L., Danguleva-Cholakova A., Todorova T., Zhelezova G., Karaivanova E., Georgieva R. (2024). In Vitro Inhibitory Effects and Co-Aggregation Activity of Lactobacilli on *Candida albicans*. Microbiol. Res..

[8] Xu Z., Li Y., Xu A., Xue L., Soteyome T., Yuan L., Ma Q., Seneviratne G., Hong W., Mao Y. (2024). Differential Alteration in *Lactiplantibacillus Plantarum* Subsp. *Plantarum* Quorum-Sensing Systems and Reduced *Candida albicans* Yeast Survival and Virulence Gene Expression in Dual-Species Interaction. Microbiol. Spectr..

[9] Lau L.Y.J., Quek S.Y. (2024). Probiotics: Health Benefits, Food Application, and Colonization in the Human Gastrointestinal Tract. Food Bioeng..

[10] Vazquez-Munoz R., Thompson A., Sobue T., Dongari-Bagtzoglou A. (2024). *Lactobacillus johnsonii* Is a Dominant *Lactobacillus* in the Murine Oral Mucosa and Has Chitinase Activity That Compromises Fungal Cell Wall Integrity. mBio.

[11] Zangl I., Pap I.-J., Aspöck C., Schüller C. (2020). The Role of *Lactobacillus* Species in the Control of *Candida* via Biotrophic Interactions. Microb. Cell.

[12] MacAlpine J., Daniel-Ivad M., Liu Z., Yano J., Revie N.M., Todd R.T., Stogios P.J., Sanchez H., O’Meara T.R., Tompkins T.A. (2021). A Small Molecule Produced by *Lactobacillus* Species Blocks *Candida albicans* Filamentation by Inhibiting a DYRK1-Family Kinase. Nat. Commun..

[13] DiMattia Z., Damani J.J., Van Syoc E., Rogers C.J. (2024). Effect of Probiotic Supplementation on Intestinal Permeability in Overweight and Obesity: A Systematic Review of Randomized Controlled Trials and Animal Studies. Adv. Nutr..

[14] Rosati D., Valentine M., Bruno M., Pradhan A., Dietschmann A., Jaeger M., Leaves I., Van De Veerdonk F.L., Joosten L.A.B., Roy S. (2025). Lactic Acid in the Vaginal Milieu Modulates the *Candida* -Host Interaction. Virulence.

[15] Food and Agriculture Organization of the United Nations, World Health Organization (2016). Statistical Aspects of Microbiological Criteria Related to Foods: A Risk Managers Guide.

[16] Atanasov N., Evstatieva Y., Nikolova D. (2023). Antagonistic Interactions of Lactic Acid Bacteria from Human Oral Microbiome against Streptococcus Mutans and *Candida albicans*. Microorganisms.

[17] Ribeiro F.C., Rossoni R.D., Barros P.P., Santos J.D., Fugisaki L.R.O., Leão M.P.V., Junqueira J.C. (2020). Action Mechanisms of Probiotics on *Candida* Spp. and Candidiasis Prevention: An Update. J. Appl. Microbiol..

[18] Robergs R., O’Malley B., Torrens S., Siegler J. (2023). The Missing Hydrogen Ion, Part-1: Historical Precedents vs. Fundamental Concepts. Sports Med. Health Sci..

[19] Davis D. (2003). Adaptation to Environmental pH in *Candida albicans* and Its Relation to Pathogenesis. Curr. Genet..

[20] Porta A., Ramon A.M., Fonzi W.A. (1999). *PRR1*, a Homolog of *Aspergillus Nidulans palF*, Controls pH-Dependent Gene Expression and Filamentation in *Candida albicans*. J. Bacteriol..

[21] Vylkova S., Lorenz M.C. (2014). Modulation of Phagosomal pH *by Candida albicans* Promotes Hyphal Morphogenesis and Requires Stp2p, a Regulator of Amino Acid Transport. PLoS Pathog..

[22] Miao H., Liang J., Lan G., Wu Q., Huang Z. (2024). Heat-Killed *Lactobacillus acidophilus* Promotes Growth by Modulating the Gut Microbiota Composition and Fecal Metabolites of Piglets. Animals.

[23] Van V.T.H., Liu Z.-S., Hsieh Y.J., Shiu W.-C., Chen B.-Y., Ku Y.-W., Chen P.-W. (2023). Therapeutic Effects of Orally Administration of Viable and Inactivated Probiotic Strains against Murine Urinary Tract Infection. J. Food Drug Anal..

[24] De Gregorio P.R., Silva J.A., Marchesi A., Nader-Macías M.E.F. (2019). Anti-*Candida* Activity of Beneficial Vaginal Lactobacilli in in Vitro Assays and in a Murine Experimental Model. FEMS Yeast Res..

[25] König H., Unden G., Fröhlich J. (2017). Biology of Microorganisms on Grapes, in Must and in Wine.

[26] Nasrollahzadeh A., Mokhtari S., Khomeiri M., Saris P.E.J. (2022). Antifungal Preservation of Food by Lactic Acid Bacteria. Foods.

[27] Hossain T.J. (2024). Methods for Screening and Evaluation of Antimicrobial Activity: A Review of Protocols, Advantages, and Limitations. EuJMI.

[28] Hoover D.G., Harlander S.K. (1993). Screening Methods for Detecting Bacteriocin Activity. Bacteriocins of Lactic Acid Bacteria.

[29] Zeng Y., Fadaak A., Alomeir N., Wu T.T., Rustchenko E., Qing S., Bao J., Gilbert C., Xiao J. (2022). *Lactobacillus plantarum* Disrupts S. Mutans–C. Albicans Cross-Kingdom Biofilms. Front. Cell. Infect. Microbiol..

[30] Bakhshi M., Salari S., Almani P.G.N., Afshari S.A.K. (2021). Evaluation of the Antifungal Activity of *Lactobacillus reuteri* against *Candida* Species. Gene Rep..

[31] Matsubara V.H., Bandara H.M.H.N., Mayer M.P.A., Samaranayake L.P. (2016). Probiotics as Antifungals in Mucosal Candidiasis. Clin. Infect. Dis..

[32] Lourenço A., Pedro N.A., Salazar S.B., Mira N.P. (2019). Effect of Acetic Acid and Lactic Acid at Low pH in Growth and Azole Resistance of *Candida albicans* and *Candida glabrata*. Front. Microbiol..

[33] Cottier F., Hall R.A. (2020). Face/Off: The Interchangeable Side of *Candida albicans*. Front. Cell. Infect. Microbiol..

[34] Mailänder-Sánchez D., Braunsdorf C., Grumaz C., Müller C., Lorenz S., Stevens P., Wagener J., Hebecker B., Hube B., Bracher F. (2017). Antifungal Defense of Probiotic *Lactobacillus rhamnosus* GG Is Mediated by Blocking Adhesion and Nutrient Depletion. PLoS ONE.

[35] Palomino M.M., Allievi M.C., Gordillo T.B., Bockor S.S., Fina Martin J., Ruzal S.M. (2023). Surface Layer Proteins in Species of the Family *Lactobacillaceae*. Microb. Biotechnol..

[36] Scillato M., Spitale A., Mongelli G., Privitera G.F., Mangano K., Cianci A., Stefani S., Santagati M. (2021). Antimicrobial Properties of *Lactobacillus* Cell-free Supernatants against Multidrug-resistant Urogenital Pathogens. MicrobiologyOpen.

[37] Behera B., Anil Vishnu G.K., Chatterjee S., Sitaramgupta V.S.N., Sreekumar N., Nagabhushan A., Rajendran N., Prathik B.H., Pandya H.J. (2019). Emerging Technologies for Antibiotic Susceptibility Testing. Biosens. Bioelectron..

[38] Robinson T.P., Wimpenny J.W.T., Earnshaw R.G. (1991). pH Gradients through Colonies of *Bacillus cereus* and the Surrounding Agar. J. Gen. Microbiol..

[39] Prakash O., Waghmare U., Chauhan A., Patil Y. (2025). Optimizing Experimental Conditions: The Role of Buffered Environments in Microbial Isolation, Physiological Studies, and Taxonomic Characterization. Appl. Env. Microbiol..

[40] Malakar P.K., Zwietering M.H., Boom R.M., Brocklehurst T.F., Wilson P.D.G., Mackie A.R., Van ’T Riet K. (2002). Diffusion of Lactic Acid in a Buffered Gel System Supporting Growth of *Lactobacillus curvatus*. J. Sci. Food Agric..

[41] Salari S., Ghasemi Nejad Almani P. (2020). Antifungal Effects of *Lactobacillus acidophilus* and *Lactobacillus plantarum* against Different Oral *Candida* Species Isolated from HIV/ AIDS Patients: An in Vitro Study. J. Oral. Microbiol..

[42] Meng L., Li S., Liu G., Fan X., Qiao Y., Zhang A., Lin Y., Zhao X., Huang K., Feng Z. (2021). The Nutrient Requirements of *Lactobacillus acidophilus* LA-5 and Their Application to Fermented Milk. J. Dairy. Sci..

[43] Alp D. (2022). Strain-dependent Effectivity, and Protective Role against Enzymes of S-layers in *Lactiplantibacillus plantarum* Strains. J. Basic. Microbiol..

[44] Angelescu I.-R., Zamfir M., Ionetic E.-C., Grosu-Tudor S.-S. (2024). The Biological Role of the S-Layer Produced by *Lactobacillus helveticus* 34.9 in Cell Protection and Its Probiotic Properties. Fermentation.

[45] Gordillo T.B., Palumbo M.C., Allievi M.C., Fernández Do Porto D.A., Ruzal S.M., Palomino M.M. (2020). Strategies to Display Heterologous Proteins on the Cell Surface of Lactic Acid Bacteria Using as Anchor the C-Terminal Domain of *Lactobacillus acidophilus* SlpA. World J. Microbiol. Biotechnol..

[46] García-Gamboa R., Domínguez-Simi M., Gradilla-Hernández M.S., Bravo J., Moya A., Ruiz-Álvarez B., González-Avila M. (2022). Anticandidal and Antibiofilm Effect of Synbiotics Including Probiotics and Inulin-Type Fructans. Antibiotics.

[47] Romera D., Aguilera-Correa J.-J., García-Coca M., Mahillo-Fernández I., Viñuela-Sandoval L., García-Rodríguez J., Esteban J. (2020). The *Galleria mellonella* Infection Model as a System to Investigate the Virulence of *Candida auris* Strains. Pathog. Dis..

[48] Thewes S., Moran G.P., Magee B.B., Schaller M., Sullivan D.J., Hube B. (2008). Phenotypic Screening, Transcriptional Profiling, and Comparative Genomic Analysis of an Invasive and Non-Invasive Strain of *Candida albicans*. BMC Microbiol..

[49] Takano T., Kudo H., Eguchi S., Matsumoto A., Oka K., Yamasaki Y., Takahashi M., Koshikawa T., Takemura H., Yamagishi Y. (2023). Inhibitory Effects of Vaginal Lactobacilli on *Candida albicans* Growth, Hyphal Formation, Biofilm Development, and Epithelial Cell Adhesion. Front. Cell. Infect. Microbiol..

[50] Graf K., Last A., Gratz R., Allert S., Linde S., Westermann M., Gröger M., Mosig A.S., Gresnigt M.S., Hube B. (2019). Keeping *Candida* Commensal: How Lactobacilli Antagonize Pathogenicity of *Candida albicans* in an in Vitro Gut Model. Dis. Models Mech..

[51] Zeise K.D., Woods R.J., Huffnagle G.B. (2021). Interplay between *Candida albicans* and Lactic Acid Bacteria in the Gastrointestinal Tract: Impact on Colonization Resistance, Microbial Carriage, Opportunistic Infection, and Host Immunity. Clin. Microbiol. Rev..

[52] Rajão A., Silva J.P.N., Almeida-Nunes D.L., Rompante P., Rodrigues C.F., Andrade J.C. (2025). *Limosilactobacillus reuteri* AJCR4: A Potential Probiotic in the Fight Against Oral *Candida* Spp. Biofilms. Int. J. Mol. Sci..

[53] Rajasekharan S.K., Venugopal A., Hameed H.C., Jacob J., Ravichandran V., Steinberg D., Faigenboim A., Raorane C.J., Shemesh M. (2025). Transcriptomic and Metabolomic Insights from Functionalized V-Shaped Lactiplantibacillus Plantarum towards Mitigating *Candida albicans* Virulence. Biofilm.

[54] Hu T., Meng Y., Zhao C., Sheng D., Yang S., Dai J., Wei T., Zhang Y., Zhao G., Liu Y. (2025). Genome-Scale Metabolic Modeling Reveals Specific Vaginal *Lactobacillus* Strains and Their Metabolites as Key Inhibitors of *Candida albicans*. Microbiol. Spectr..

[55] Vilela S.F., Barbosa J.O., Rossoni R.D., Santos J.D., Prata M.C., Anbinder A.L., Jorge A.O., Junqueira J.C. (2015). *Lactobacillus acidophilus* ATCC 4356 Inhibits Biofilm Formation by *C. albicans* and Attenuates the Experimental Candidiasis in *Galleria mellonella*. Virulence.

[56] Klarin B., Molin G., Jeppsson B., Larsson A. (2008). Use of the Probiotic *Lactobacillus plantarum* 299 to Reduce Pathogenic Bacteria in the Oropharynx of Intubated Patients: A Randomised Controlled Open Pilot Study. Crit. Care.

[57] Putonti C., Shapiro J.W., Ene A., Tsibere O., Wolfe A.J. (2020). Comparative Genomic Study of *Lactobacillus jensenii* and the Newly Defined *Lactobacillus mulieris* Species Identifies Species-Specific Functionality. mSphere.

[58] Bnfaga A.A., Lee K.W., Than L.T.L., Amin-Nordin S. (2023). Antimicrobial and Immunoregulatory Effects of *Lactobacillus delbrueckii* 45E against Genitourinary Pathogens. J. Biomed. Sci..

[59] Bao J., Huang X., Zeng Y., Wu T.T., Lu X., Meng G., Ren Y., Xiao J. (2023). Dose-Dependent Inhibitory Effect of Probiotic *Lactobacillus plantarum* on Streptococcus Mutans-*Candida albicans* Cross-Kingdom Microorganisms. Pathogens.

