# Supplementary material for: Probiotic and Postbiotic Interactions of Lactobacillus Strains with Candida albicans: Antifungal Effects Through Microbial Competition
Source: Antibiotics (Basel). 2026 Mar 10;15(3):279. doi: 10.3390/antibiotics15030279 (PMC13023605; doi:10.3390/antibiotics15030279)
Supplement: Supplementary file 1 [file antibiotics-15-00279-s001.zip › antibiotics-4136979-supplementary.pdf]

Probiotic and Postbiotic Interactions of Lactobacillus Strains with *Candida albicans*: Antifungal Effects Through Microbial Competition

Andrea Vega-Vásconez[1,2], Diana Lucinda Castillo-Patiño[1,2], Javier Alberto Garza-Cervantes[1,2], Arlette Santacruz[3], José Rubén Morones-Ramírez[1,2*]

[1] Facultad de Ciencias Químicas, Universidad Autónoma de Nuevo León, San Nicolás de los Garza 66455, México.

[2] Centro de Investigación en Biotecnología y Nanotecnología, Facultad de Ciencias Químicas, Universidad Autónoma de Nuevo León, Parque de Investigación e Innovación Tecnológica, Apodaca 66628, México

[3] School of Engineering and Science, Tecnologico de Monterrey, Av. Eugenio Garza Sada 2501 Sur, Monterrey C.P. 64849, Nuevo León, Mexico

*Correspondence: José Rubén, Morones-Ramírez, jose.moronesrmr@uanl.edu.mx

A

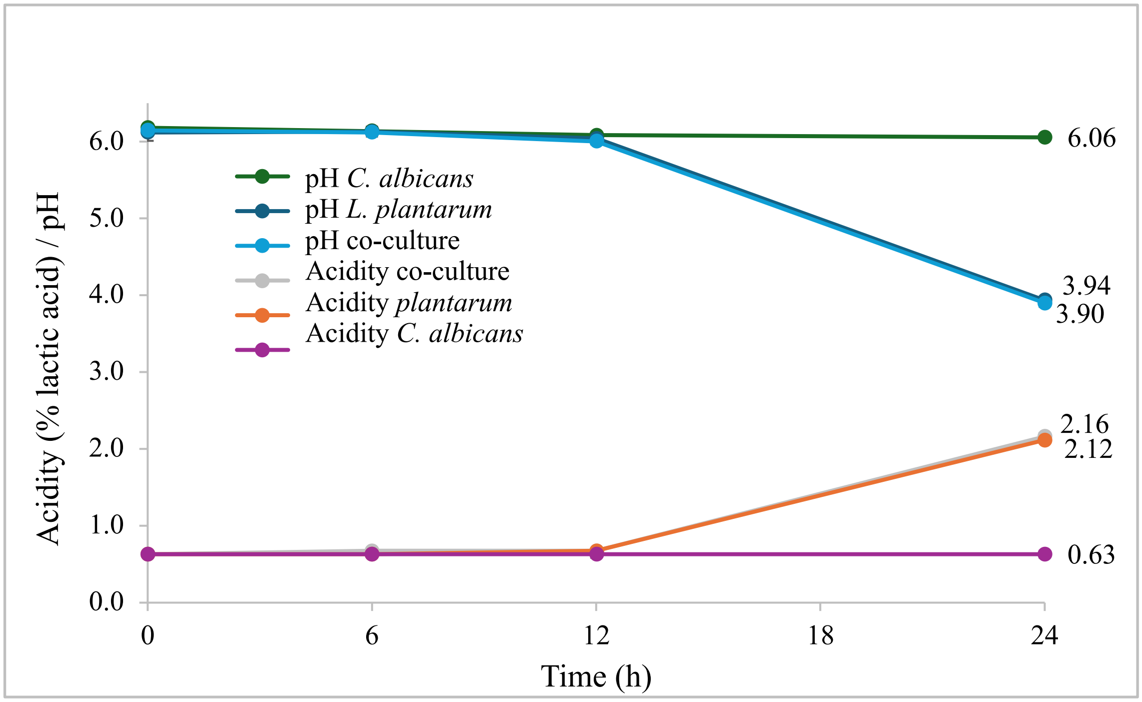

B

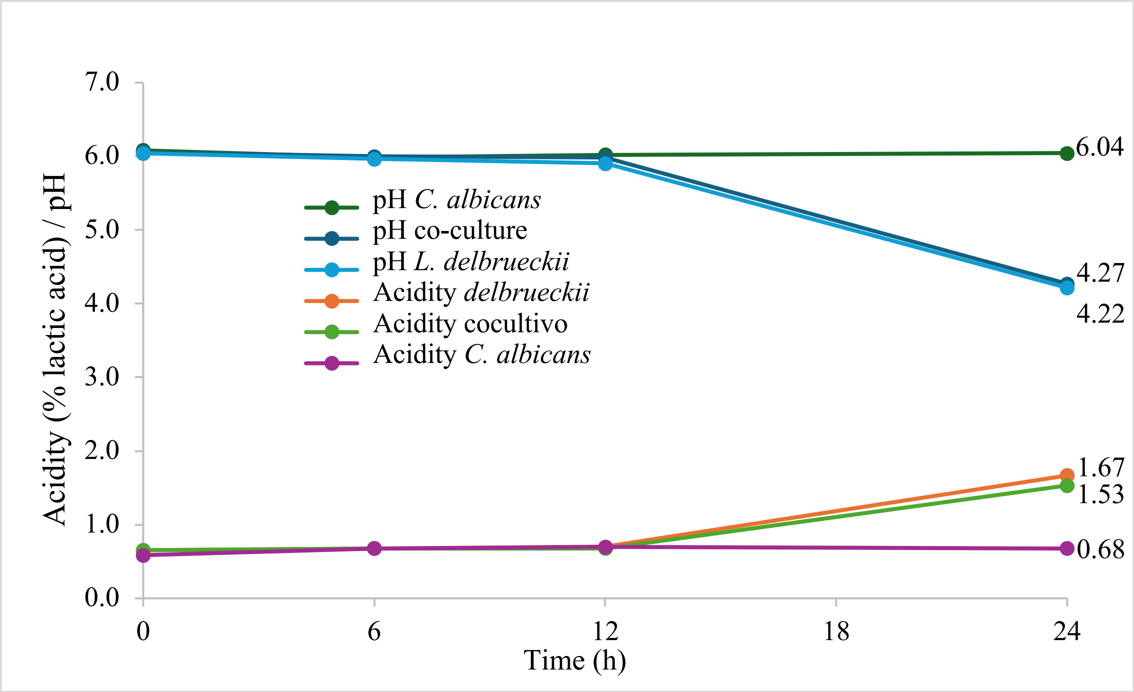

7.0
6.0
5.0
4.0
3.0
2.0
1.0
0.0
Acidity (% lactic acid) / pH
pH C. albicans
pH co-culture
pH L. delbrueckii
Acidity delbrueckii
Acidity cocultivo
Acidity C. albicans
6.04
4.27
4.22
1.67
1.53
0.68
0
6
12
18
24
Time (h)

C

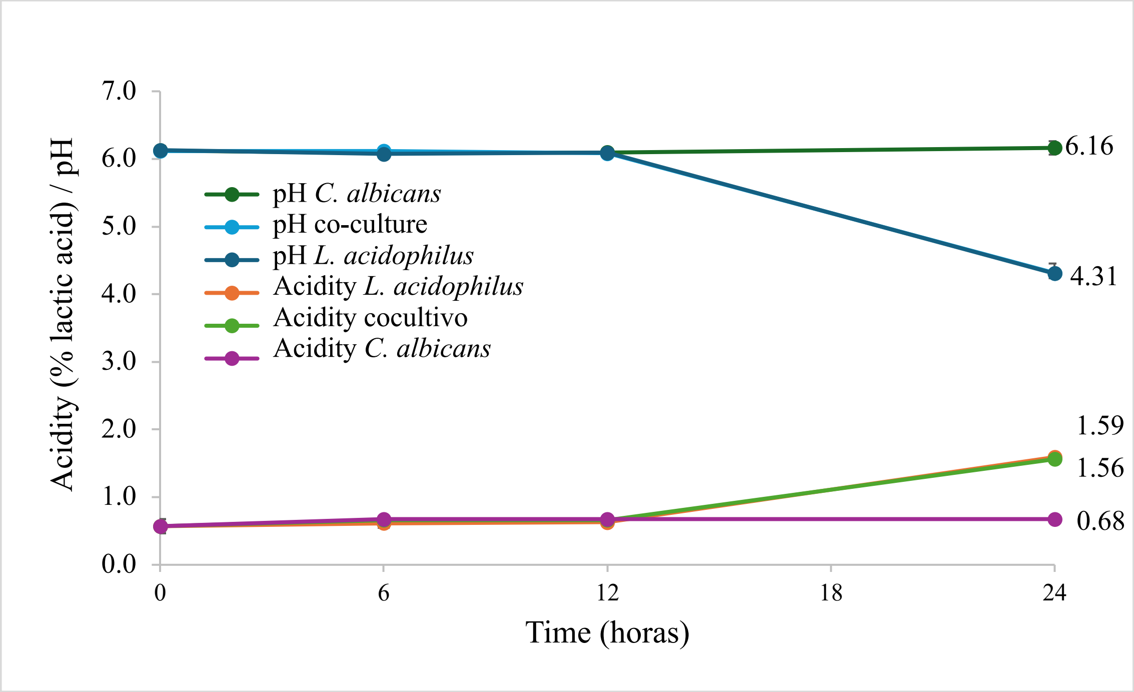

7.0
6.0
5.0
4.0
3.0
2.0
1.0
0.0
Acidity (% lactic acid) / pH
pH C. albicans
pH co-culture
pH L. acidophilus
Acidity L. acidophilus
Acidity cocultivo
Acidity C. albicans
6.16
4.31
1.59
1.56
0.68
0
6
12
18
24
Time (horas)

Figure S1. Acidity (% lactic acid) and pH of *C. albicans* vs. BAL. (A) *L. plantarum vs C. albicans* B) *L. delbrueckii vs C. albicans* C) *L. acidophilus vs C. albicans*

Table S1. *Coculture of C. albicans and L. plantarum, L. delbrueckii, L. acidophilus*

Coculture	Microorganism
1	*C. albicans* 1 × 10^2 UFC mL^{-1} + *L. plantarum* 1 × 10^3 UFC mL^{-1}
2	*C. albicans* 1 × 10^2 UFC mL^{-1} + *L. delbrueckii* 1 × 10^3 UFC mL^{-1}
3	*C. albicans* 1 × 10^2 UFC mL^{-1} + *L. acidophilus* 1 × 10^3 UFC mL^{-1}

Table S2. Components of the Supplemented MRS

Component	Quantity (g/L)
MRS Broth	55.0 g
Peptone	0.5 g
Yeast Extract	0.25 g
